# Structural and electrophysiological determinants of atrial cardiomyopathy identify remodeling discrepancies between paroxysmal and persistent atrial fibrillation

**DOI:** 10.3389/fcvm.2022.1101152

**Published:** 2023-01-11

**Authors:** Taiyuan Huang, Deborah Nairn, Juan Chen, Bjoern Mueller-Edenborn, Nicolas Pilia, Louisa Mayer, Martin Eichenlaub, Zoraida Moreno-Weidmann, Juergen Allgeier, Dietmar Trenk, Christoph Ahlgrim, Dirk Westermann, Thomas Arentz, Axel Loewe, Amir Jadidi

**Affiliations:** ^1^Arrhythmia Division, Department of Cardiology, Faculty of Medicine, University Heart Center Freiburg-Bad Krozingen, University of Freiburg, Freiburg im Breisgau, Germany; ^2^Institute of Biomedical Engineering (IBT), Karlsruhe Institute of Technology (KIT), Karlsruhe, Germany; ^3^Department of Cardiology, The Affiliated Drum Tower Hospital, Medical School of Nanjing University, Nanjing, China

**Keywords:** atrial fibrillation, atrial cardiomyopathy, mapping, ECG, machine learning

## Abstract

**Background:**

Progressive atrial fibrotic remodeling has been reported to be associated with atrial cardiomyopathy (ACM) and the transition from paroxysmal to persistent atrial fibrillation (AF). We sought to identify the anatomical/structural and electrophysiological factors involved in atrial remodeling that promote AF persistency.

**Methods:**

Consecutive patients with paroxysmal (*n* = 134) or persistent (*n* = 136) AF who presented for their first AF ablation procedure were included. Patients underwent left atrial (LA) high-definition mapping (1,835 ± 421 sites/map) during sinus rhythm (SR) and were randomized to training and validation sets for model development and evaluation. A total of 62 parameters from both electro-anatomical mapping and non-invasive baseline data were extracted encompassing four main categories: (1) LA size, (2) extent of low-voltage-substrate (LVS), (3) LA voltages and (4) bi-atrial conduction time as identified by the duration of amplified P-wave (APWD) in a digital 12-lead-ECG. Least absolute shrinkage and selection operator (LASSO) and logistic regression were performed to identify the factors that are most relevant to AF persistency in each category alone and all categories combined. The performance of the developed models for diagnosis of AF persistency was validated regarding discrimination, calibration and clinical usefulness. In addition, HATCH score and C2HEST score were also evaluated for their performance in identification of AF persistency.

**Results:**

In training and validation sets, APWD (threshold 151 ms), LA volume (LAV, threshold 94 mL), bipolar LVS area < 1.0 mV (threshold 4.55 cm^2^) and LA global mean voltage (GMV, threshold 1.66 mV) were identified as best determinants for AF persistency in the respective category. Moreover, APWD (AUC 0.851 and 0.801) and LA volume (AUC 0.788 and 0.741) achieved better discrimination between AF types than LVS extent (AUC 0.783 and 0.682) and GMV (AUC 0.751 and 0.707). The integrated model (combining APWD and LAV) yielded the best discrimination performance between AF types (AUC 0.876 in training set and 0.830 in validation set). In contrast, HATCH score and C2HEST score only achieved AUC < 0.60 in identifying individuals with persistent AF in current study.

**Conclusion:**

Among 62 electro-anatomical parameters, we identified APWD, LA volume, LVS extent, and mean LA voltage as the four determinant electrophysiological and structural factors that are most relevant for AF persistency. Notably, the combination of APWD with LA volume enabled discrimination between paroxysmal and persistent AF with high accuracy, emphasizing their importance as underlying substrate of persistent AF.

## 1. Introduction

Atrial fibrillation (AF) is the most common supraventricular arrhythmia in humans and is associated with an increased risk for cardiovascular complications as ischemic stroke, heart failure and mortality (1–5). Although, previous clinical studies have revealed that structural (atrial dilation, atrial fibrotic remodeling) and electrical remodeling of the atrial myocardium is associated with development of AF, the importance and contribution of each factor is not thoroughly studied (6–8). Moreover, the pathophysiological processes responsible for persistency of AF are incompletely understood (9). In an effort to better predict progression from paroxysmal to persistent AF, the HATCH score, which consists of different clinical parameters, was developed and validated in various studies (10). Another important risk estimation tool, the C2HEST score, which was developed to predict incident AF also shared similar components and was widely validated in different clinical studies (11). Nevertheless, both HATCH score and C2HEST score, despite their original purposes of prediction of future events, were not evaluated regarding their performance in identification of AF persistency among AF patients. Atrial remodeling, which has been identified as a major contributor to arrhythmogenesis in persistent AF, was associated with pathological manifestations both structurally and electrophysiologically (12, 13). Given that both HATCH score and C2HEST score do not include information on atrial remodeling, we hypothesize that a new model that reflects the underlying atrial remodeling processes may be more accurate to differentiate between paroxysmal and persistent AF and identify persons at risk for progression to persistent AF type. The aim of the current study is to identify the major structural (left atrial (LA) volume (LAV), LA surface area) and electrophysiological factors [global LA voltage, extent of LA low voltage substrate (LVS), and bi-atrial conduction time as identified by the 12-lead-ECG-derived duration of the amplified digital P-wave (APWD)] that are associated with atrial cardiomyopathy (ACM) and development of persistent AF. Therefore, we assessed 62 parameters from LA high-density voltage maps and non-invasive parameters including ECG recorded during sinus rhythm (SR) in 270 patients with AF.

## 2. Materials and methods

### 2.1. Study population

Consecutive patients with confirmed diagnosis of paroxysmal (<7 days AF duration) or persistent AF (>7 days and < 12 months AF duration or sustained AF necessitating electrical cardioversion to SR) referred to our heart center between 2017 and 2022 for first pulmonary vein isolation (PVI) were prospectively recruited for the current study. All patients underwent 12-lead ECG after admission. Exclusion criteria were contra-indications for PVI: presence of LA thrombus, advanced malignancy, overt clinical hyperthyroidism [elevated triiodothyronine (T3), thyroxine (T4), and suppressed thyroid stimulating hormone (TSH)], prior catheter ablation therapy or prior cardiac surgery. As a result, 270 individuals were allocated into the paroxysmal AF cohort (*n* = 134) or the persistent AF cohort (*n* = 136). Additionally, the entire study cohort was further randomized into training set and validation set with a predefined ratio of 7:3 for model development and evaluation. The study was approved by the institutional ethics committee and all patients provided written informed consent prior to enrolment.

### 2.2. Digital 12-lead ECG recording and processing

The SR ECG of all participants prior to PVI was recorded using the LabsystemPro EP-system (Boston Scientific) with the following filter settings: 0.05–100 Hz at a sampling rate of 1,000 Hz. A 10 s interval of each ECG was exported for further measurement using amplified scaling.

**FIGURE 1:**
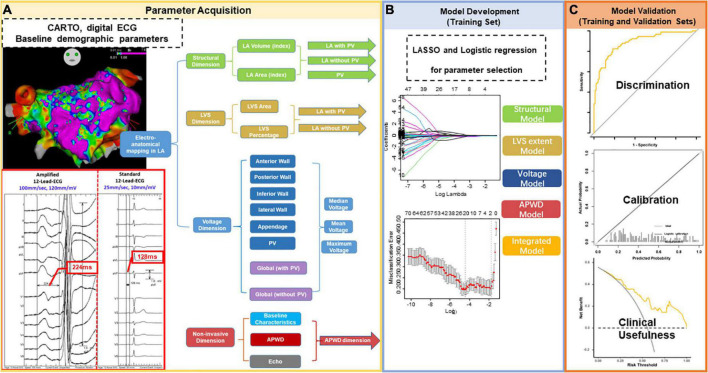
Study Flowchart. The study comprised three major steps. Step 1 for parameter acquisition **(A)** was performed in all enrolled participants with extraction of data from LA electro-anatomical mapping (LA structure, LVS extent, and LA voltage) and non-invasive parameters (ECG and clinical baseline characteristics, echocardiography). Extracted data were categorized into four categories/dimensions [**(A)**: structural dimension (green), LVS dimension (brown), voltage dimension (blue) and non-invasive parameters (red)]. The measurement of APWD in comparison to standard PWD from the same ECG at the same beat is illustrated in the red box in **(A)**. In contrast to standard setting (scale at 10 mm/mV and sweeping speed at 25 mm/s), the duration of P-wave in the current study (APWD) is measured at an amplified scale (60–120 mm/mV and 100 mm/s), which enables an improved accuracy in quantifying bi-atrial conduction time. Step 2: model development **(B)** was performed in the training set using LASSO and logistic regression for selecting the most relevant parameters for AF-persistency from each of the four different categories/dimensions. The selected parameters were used to construct diagnostic models and to undergo model validation [Step 3 in **(C)**] in both training and validation sets. LA, left atrial; LVS, low voltage substrate; ECG, electrocardiogram; APWD, duration of the amplified digital P-wave; LASSO, least absolute shrinkage and selection operator.

We previously reported a novel ECG analysis method using amplified scaling to measure P-wave duration (PWD) (14, 15). In contrast to conventional standard scaling (scale at 10 mm/mV and weeping speed at 25 mm/s), we amplified the voltage amplitude and sweeping speed to 60–120 mm/mV and 100–200 mm/s to obtain optimal signal-to-noise ratio for good visualization of P-wave beginning and ending (Figure 1). The duration of the amplified P-wave (APWD) (from the earliest P-wave onset until latest P-wave ending in any of the 12 leads at high amplification scaling) was subsequently measured using digital calipers by two independent cardiologists who were blinded to patient characteristics. Advanced inter-atrial block (aIAB) was defined as an initially positive P-wave with negative terminal deflection in at least two of the three inferior leads.

### 2.3. Electro-anatomical mapping

As illustrated in Figure 1, high-density intra-cardiac voltage and activation mapping of the left atrium (LA) was performed in all individuals during SR as described previously using the CARTO-3 mapping system (Biosense Webster, Diamond Bar, 35 CA, USA) and a 20-pole Lasso-Nav catheter (electrode size: 1 mm, spacing: 2–6–2 mm) (16). Additionally, the mitral annulus was manually removed from each bipolar map. Anatomical regions were defined on the patient-specific geometry (Supplementary Figure 1). Peak-to-peak voltage values from each mapped site within each LA region were extracted from maps for further regional and global analysis.

### 2.4. Analysis of electro-anatomical and electrocardiographic data

In order to identify the determinant parameters involved in persistency of AF, a total of 62 parameters were extracted: 47 from the LA electro-anatomical maps and 15 non-invasive baseline parameters including APWD from the digital 12-lead-ECG (Figure 1). Extracted data were attributed to four categories (also called “dimensions”) encompassing (1) LA structural remodeling (“structural dimension”), (2) global and regional voltage (“voltage dimension”), (3) LVS extent (“LVS dimension”), and (4) non-invasive parameters (including bi-atrial conduction time as identified by APWD in the 12-lead-ECG, baseline characteristics and echocardiographic measurements).

#### 2.4.1. Assessment of left atrial structural remodeling (LA structural dimension)

As shown in Figure 1, the extent of LA structural remodeling “structural dimension” was evaluated using left atrial volume (LAV) and LA surface area that were obtained from electro-anatomical mapping data. LAV index (LAVI) and LA area index were calculated as the ratio of LAV and LA area divided by the patient-specific body surface area (BSA). In order to account for the role of the pulmonary veins (PV) in structural remodeling, the volume and surface area of the PVs were also derived from the electro-anatomical map. Additionally, the aforementioned parameters were stratified into three subgroups (1) LA with PVs, (2) LA without PVs and (3) PVs, in order to provide comprehensive information of LA structural dimension.

#### 2.4.2. Assessment of left atrial low voltage substrate (LVS dimension)

The extent of LVS was quantified by (1) measurement of the absolute surface area (cm^2^) of low voltage areas in SR map using bipolar thresholds of < 0.5 and < 1.0 mV, respectively. (2) The percentage of LVS calculated as the ratio of LVS area (cm^2^) divided by total LA surface area (after exclusion of mitral valve surface areas). Consistent with the structural dimension, subgroups were also provided for the LVS dimension by inclusion or exclusion of PVs, as illustrated in Figure 1.

#### 2.4.3. Assessment of left atrial voltage (LA voltage dimension)

Based on the bipolar map during SR (Supplementary Figure 1), the LA was divided into (1) anterior wall, (2) posterior wall, (3) inferior wall, (4) lateral wall, (5) left atrial appendage (LAA), and (6) PVs. Subsequently, voltages were recorded and analyzed both globally for the entire LA and regionally for each segment. Similar to the “structural dimension,” global voltage was stratified by subgroups of “LA with PVs” and “LA without PVs” Furthermore, both global and regional voltage values were calculated, respectively, as mean, median and maximum values (Figure 1). We used the following definitions to define PV ostia and antra, respectively: PV ostium was defined as the point of maximal inflection between the PV wall and the LA wall (17, 18). Following a previous classification by Rodriguez-Manero et al., PV antrum was defined as the 5 mm LA wall that is located beyond the PV ostium (Supplementary Figure 1) (19).

#### 2.4.4. Non-invasive dimension

Apart from the aforementioned three dimensions that consisted of parameters derived from endocardial LA electro-anatomical mapping, we pooled data from other 15 non-invasive parameters to establish a non-invasive dimension, which included the bi-atrial conduction time was determined from the digital 12-lead-ECG by measurement of APWD as described in previous section “2.2. Digital 12-lead ECG recording and processing.” The other 14 non-invasive parameters from baseline demographic characteristics included age, gender, body mass index (BMI), BSA, LA diameter (LAD), left ventricular ejection fraction (LVEF), CHA2DS2-VASc score, presence of aIAB, heart failure, hypertension, diabetes, coronary artery disease (CAD), stroke and transient ischemic attack (TIA).

### 2.5. HATCH score and C2HEST score

The detailed components and corresponding score of the two scoring system were reported previously. Based on the baseline characteristics of enrolled patients, the HATCH score and C2HEST score of each individual were computed. Moreover, as the original cut-off points were developed to predict future events whereas the current study focused on detection of AF persistency at current circumstances, the scores were reported directly without further stratification into high or low risk subgroups.

## 3. Statistical analysis

Continuous variables were expressed as mean ± SD or median ± interquartile range based upon distribution status, and comparisons between two cohorts was performed using *t*-test or Mann-Whitney *U*-test. Categorical variables were expressed as frequency and percentage (%) and were compared by Chi-square test or Fisher’s exact test.

### 3.1. Machine learning model development

Given the aforementioned study design, we intended to develop models that focused on the remodeling discrepancies between paroxysmal and persistent AF cohorts from the different dimensions using data of the training set. Considering the underlying collinearity that existed in various parameters from voltage mapping, establishing models with all available parameters in each dimension carries high risk of over-fitting. Therefore, we used the least absolute shrinkage and selection operator (LASSO) regression approach to remove irrelevant and redundant factors in different dimensions (Figure 1). LASSO is a regularization method that reduces collinearity by introducing a penalty coefficient (λ) into the regression equation. As the coefficients of certain variables are gradually compressed to zero, only the most relevant variables for the outcome are retained, thus achieving the goal of dimensionality reduction and minimizing the risk of overfitting. Tuning hyperparameter (λ) selection in the LASSO model was performed using “cv. glmnent” functions with 10-fold cross-validation to compute the misclassification error. The function runs glmnet nfolds + 1 times, the first to get the lambda sequence, and then the remainder to compute the fit with each of the folds omitted. The error is accumulated, and the average error and standard deviation over the folds is computed (20). Subsequently, predictors selected by LASSO regression underwent multivariate logistic regression to further determine the significant parameters (*p* < 0.05) that best identify persistent AF patients in the respective dimension. After the development of four single-dimension diagnostic models (structural model, LVS extent model, voltage model and APWD model) for identifying individuals with persistent AF, an integrated model was also established using LASSO and logistic regression to identify significant variables from all candidate parameters combined (62 both invasive and non-invasive parameters).

### 3.2. Machine learning model validation

Evaluation of models was performed both in the training set and the validation set regarding their efficacy in discrimination, calibration, net benefit and diagnostic performance for identifying individuals with persistent AF (Figure 1).

Discriminative power of each model for identifying persistent AF patients was quantified by area under the curve (AUC) of the respective receiver operating characteristics (ROC) curve, ranging from 0.5 (random forecast) to 1.0 (perfect discrimination). Net reclassification improvement (NRI) is an index that quantifies how well a new model reclassifies subjects—either appropriately or inappropriately—as compared to an old model. Integrated discrimination improvement (IDI) is a statistical parameter to estimate the incremental discriminative power between models.

After the components of each model were determined, the individual probability for persistent AF by each model was estimated. A calibration plot of each model visualizes the agreement between estimated probabilities for persistent AF by diagnostic models and the actual probabilities observed in each set. Moreover, Brier score (0 for total accuracy, 1 for wholly inaccurate) was computed to assess the calibration performance of each model.

The net benefit of selected models across a range of probability threshold was illustrated by decision curve analysis (DCA). The “None” and “All” curve indicated the expected net benefit when intervention was performed to “none” and “all” of the patients. The model which positioned nearest to the right upper corner carried highest net benefit across the range of thresholds.

Diagnostic performance evaluation of each diagnostic model consisted of sensitivity, specificity, positive predictive value (PPV), negative predictive value (NPV) and accuracy. Based on the ROC curve of each model derived from the training set, optimal diagnostic thresholds for persistent AF identification was determined by Youden Index (sensitivity + specificity −1). The diagnostic performance of each model was subsequently evaluated in both training and validation sets using determined thresholds from the training set. Moreover, given the multivariable feature of the integrated model, a nomogram was constructed from the training set to further facilitate decision-making. The diagnostic performance of HATCH score and C2HEST score was evaluated from the minimal points (0) to maximum points (“7” for HATCH score and “8” for C2HEST score) with every one point of increment.

Statistical analysis was performed with SPSS version 27.0 for Macintosh (IBM Corporation, Armonk, NY), and R software version 4.0.3^1^ using glmnet, rms, pROC, ggplot2, rmda, ggDCA, caret, ggradar, and PredictABEL packages.

## 4. Results

### 4.1. Patient characteristics and randomization

A total of 270 patients were prospectively enrolled in our study. Consecutive patients presenting for their first PVI procedure were included in a 1:1 ratio with regard to the underlying AF type: In total, 134 (49.6%) patients presented paroxysmal AF and 136 (50.4%) persistent AF. Table 1 lists the characteristics of both cohorts. Heart failure showed a significantly higher prevalence in the persistent AF cohort. There were no differences in clinical baseline characteristics between paroxysmal and persistent AF types with regard to age (*p* = 0.143), sex (*p* = 0.987), BMI (*p* = 0.655), BSA (*p* = 0.159), or arterial hypertension (*p* = 0.063). However, electrocardiographic and echocardiographic parameters displayed significant differences between paroxysmal and persistent AF cohorts (APWD: 136.8 ± 13.9 ms vs. 160.4 ± 22.3 ms, *p* < 0.001; aIAB: 15.7 vs. 42.6%, *p* < 0.001; LAD: 39.89 ± 4.71 mm vs. 43.79 ± 5.62 mm, *p* < 0.001; LVEF: 60.05 ± 9.06% vs. 56.92 ± 11.79%, *p* = 0.016, respectively). Notably, significant differences were also observed between the two AF types with the electro-anatomical invasive parameters of LA remodeling (Table 2). Moreover, both HATCH score and C2HEST score were significantly higher in patients with persistent AF than paroxysmal AF (HATCH score: 1.52 ± 1.33 vs. 1.12 ± 1.10, *p* = 0.01; C2HEST score: 1.68 ± 1.36 vs. 1.28 ± 1.21, *p* = 0.016).

**TABLE 1:** Baseline characteristics between paroxysmal AF and persistent AF cohort.

	Paroxysmal AF (*n* = 134)	Persistent AF (*n* = 136)	*p*-value
Female, *n* (%)	58 (43.3%)	59 (43.4%)	0.987
Age, years	62.7 ± 12.0	64.7 ± 10.2	0.143
BMI, kg/m^2^	27.93 ± 4.73	28.18 ± 4.64	0.655
BSA, cm^2^	1.97 ± 0.22	2.00 ± 0.21	0.159
LAD, mm	39.89 ± 4.71	43.79 ± 5.62	<0.001
LVEF,%	60.05 ± 9.06	56.92 ± 11.79	0.016
CHA2DS2-VASc score	2.17 ± 1.64	2.58 ± 1.50	0.033
APWD, ms	136.8 ± 13.9	160.4 ± 22.3	<0.001
aIAB, *n* (%)	21 (15.7%)	58 (42.6%)	<0.001
Heart failure, *n* (%)	9 (6.7%)	40 (29.4%)	<0.001
Hypertension, *n* (%)	77 (57.5%)	93 (68.4%)	0.063
Diabetes, *n* (%)	17 (12.7%)	15 (11.0%)	0.674
Stroke, *n* (%)	2 (2.2%)	5 (3.7%)	0.736
TIA, *n* (%)	7 (5.2%)	4 (2.9%)	0.343
CAD, *n* (%)	18 (13.4%)	12 (8.8%)	0.228
COPD, *n* (%)	0	1 (0.7%)	0.241
Edoxaban, *n* (%)	17 (12.7%)	15 (11.0%)	0.674
HATCH score	1.12 ± 1.10	1.52 ± 1.33	0.010
C2HEST score	1.28 ± 1.21	1.68 ± 1.36	0.016
Dabigatran, *n* (%)	13 (9.7%)	9 (6.6%)	0.354
Rivaproxaban, *n* (%)	49 (36.6%)	61 (44.9%)	0.166
Apixaban, *n* (%)	38 (28.4%)	35 (25.7%)	0.628
Other OAC, *n* (%)	11 (8.2%)	14 (10.3%)	0.555
Amiodarone, *n* (%)	16 (11.9%)	53 (39.0%)	<0.001
Flecainid, *n* (%)	23 (17.2%)	18 (13.2%)	0.368
Beta blocker, *n* (%)	83 (61.9%)	89 (65.4%)	0.550

BMI, body mass index; BSA, body surface area; LAD, left atrial diameter; LVEF, left ventricular ejection fraction; APWD, duration of the amplified digital P-wave; aIAB, advanced inter-atrial block; TIA, transient ischemic attack; CAD, coronary artery disease; COPD, Chronic Obstructive Pulmonary Disease; OAC, oral anticoagulant.

**TABLE 2:** Electro-anatomical characteristics between paroxysmal AF cohort and persistent AF cohort.

1,835 ± 421 sites/map	Paroxysmal AF (*n* = 134)	Persistent AF (*n* = 136)	*p*-value
**LVS extent dimension (bipolar threshold of 1.0 and 0.5 mV)**			
LVS area with PV (0.5 mV), (cm^2^)	32.25 ± 18.90	43.27 ± 26.71	<0.001
LVS area without PV (0.5 mV), (cm^2^)	0.04 ± 0.46	1.43 ± 7.52	<0.001
LVS area with PV (1 mV), (cm^2^)	50.75 ± 23.50	69.42 ± 47.69	<0.001
LVS area without PV (1 mV), (cm^2^)	2.00 ± 6.58	11.69 ± 24.38	<0.001
LVS percentage without PV (0.5 mV), (%)	0.061 ± 0.88	1.72 ± 12.31	<0.001
LVS percentage with PV (0.5 mV), (%)	21.15 ± 12.48	25.89 ± 20.11	<0.001
LVS percentage without PV (1 mV), (%)	3.64 ± 11.03	16.93 ± 40.11	<0.001
LVS percentage with PV (1 mV), (%)	33.58 ± 15.39	43.57 ± 33.50	<0.001
**Structural dimension**			
LA volume with PV, (mL)	129.32 ± 24.22	157.64 ± 35.47	<0.001
LA volume without PV, (mL)	83.71 ± 18.26	105.33 ± 26.61	<0.001
LA volume index with PV, (mL/m^2^)	66.17 ± 12.25	79.10 ± 17.39	<0.001
LA volume Index without PV, (mL/m^2^)	42.85 ± 9.21	52.96 ± 13.61	<0.001
PV volume, (mL)	45.60 ± 9.22	52.31 ± 14.72	<0.001
PV volume index, (mL/m^2^)	23.32 ± 4.65	26.14 ± 6.73	<0.001
LA area with PV, (cm^2^)	150.40 ± 20.69	158.94 ± 30.70	0.008
LA area without PV, (cm^2^)	63.65 ± 10.23	71.44 ± 13.82	<0.001
LA area index with PV, (cm^2^/m^2^)	76.87 ± 9.71	79.36 ± 12.56	0.069
LA area index without PV, (cm^2^/m^2^)	32.57 ± 5.14	35.85 ± 6.75	<0.001
PV area, (cm^2^)	86.75 ± 15.57	87.49 ± 22.84	0.755
PV area index, (cm^2^/m^2^)	44.31 ± 7.30	43.52 ± 9.57	0.448
**Voltage dimension, (mV)**			
Bipolar LA global median voltage without PV	2.43 ± 0.81	1.82 ± 0.77	<0.001
Bipolar LA global mean voltage without PV	2.18 ± 0.75	1.53 ± 0.68	<0.001
Bipolar LA global mean voltage with PV	1.66 ± 0.55	1.29 ± 0.55	<0.001
Bipolar LA global median voltage with PV	1.39 ± 0.52	1.04 ± 0.52	<0.001
Bipolar LA maximum voltage without LAA	11.36 ± 3.50	9.62 ± 3.59	<0.001
Bipolar LA maximum voltage with LAA	9.08 ± 4.42	7.05 ± 3.55	<0.001
Bipolar LA anterior mean voltage	2.12 ± 0.99	1.44 ± 0.91	<0.001
Bipolar LA posterior mean voltage	2.57 ± 1.17	1.74 ± 0.80	<0.001
Bipolar LA inferior mean voltage	2.03 ± 0.78	1.57 ± 0.70	<0.001
Bipolar LA lateral mean voltage	2.53 ± 0.89	1.98 ± 0.92	<0.001
Bipolar LAA mean voltage	3.51 ± 1.42	2.72 ± 1.40	<0.001
Bipolar left PV mean voltage	0.84 ± 0.46	0.70 ± 0.50	0.019
Bipolar right PV mean voltage	1.09 ± 0.47	0.84 ± 0.45	<0.001
Bipolar LA anterior median voltage	1.96 ± 0.95	1.30 ± 0.87	<0.001
Bipolar LA posterior median voltage	2.37 ± 1.13	1.59 ± 0.97	<0.001
Bipolar LA inferior median voltage	1.83 ± 0.72	1.42 ± 0.67	<0.001
Bipolar LA lateral median voltage	2.38 ± 0.91	1.83 ± 0.93	<0.001
Bipolar LAA median voltage	3.41 ± 1.43	2.63 ± 1.40	<0.001
Bipolar left PV median voltage	0.54 ± 0.44	0.47 ± 0.45	0.237
Bipolar right PV median voltage	0.79 ± 0.46	0.62 ± 0.42	0.001
Bipolar LA anterior maximum voltage	7.36 ± 3.27	5.65 ± 3.15	<0.001
Bipolar LA posterior maximum voltage	9.11 ± 3.66	6.80 ± 3.30	<0.001
Bipolar LA inferior maximum voltage	8.53 ± 3.40	6.98 ± 2.99	<0.001
Bipolar LA maximum voltage	8.34 ± 2.91	7.01 ± 3.09	<0.001
Bipolar LAA maximum voltage	8.82 ± 3.75	7.05 ± 3.55	<0.001
Bipolar left PV maximum voltage	8.31 ± 5.00	6.86 ± 3.52	0.006
Bipolar right PV maximum voltage	8.74 ± 7.25	6.34 ± 3.21	<0.001

LVS, low voltage substrate; PV, pulmonary vein; LA, left atrial; LAA, left atrial appendage.

To identify the major structural and electrophysiological factors involved in AF persistency, we used statistical models to differentiate between both AF types. Therefore, 70% of the entire study cohort was randomized into the training set to develop statistical predictive models and internally validate their discriminative efficacy between AF subtypes. The remaining 30% formed a validation set, as in Supplementary Table 1.

### 4.2. Model development in training set

#### 4.2.1. Single-dimension models

**FIGURE 2:**
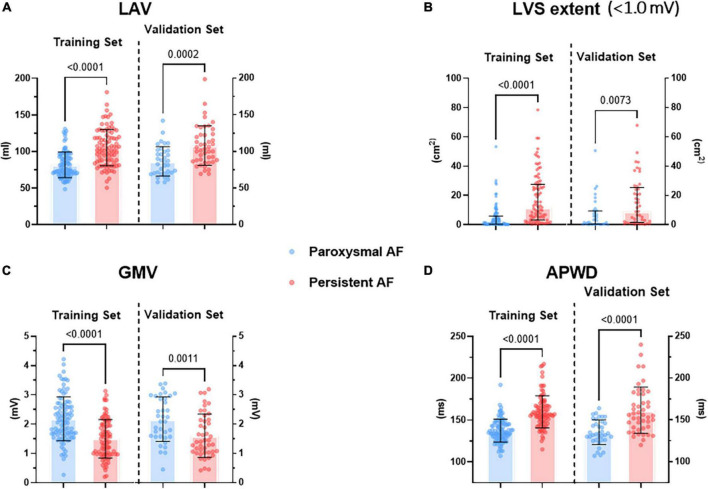
Difference in selected parameters from each dimension between paroxysmal (blue) and persistent (red) AF cohorts in both training and validation sets. **(A)** Illustrates the difference in LAV, **(B)** illustrates the difference in LVS extent at < 1.0 mV threshold during sinus rhythm, **(C)** illustrates the difference in GMV, **(D)** illustrates the difference in APWD. LAV, left atrial volume; LVS, low voltage substrate; GMV, global mean voltage; APWD, duration of the amplified digital P-wave.

Among all parameters acquired from LA high density electro-anatomical mapping, only those which were measured without inclusion of PVs were selected by LASSO regression as most discriminative for respective category/dimension. More precisely, after 10-fold cross validation of hyperparameter tuning (λ value of minimum and 1-SE criteria at 0.0438 and 0.0765, respectively. Misclassification error of 0.191 ± 0.023 and 0.206 ± 0.036 for minimum and 1-SE criteria, respectively), LASSO regression identified two parameters (LAV and LAVI) from “structural dimension,” one parameter (LVS area at 1.0 mV bipolar threshold) from “LVS dimension,” one parameter [global mean voltage (GMV)] from “voltage dimension.” Among a total of 15 non-invasive parameters, only APWD was selected, as shown in Table 3. After multivariate logistic regression in each category/dimension, only LAV (*p* = 0.031), LVS area at 1.0 mV bipolar threshold (LVS extent) (*p* < 0.001), GMV (*p* < 0.001) and APWD (*p* < 0.001) were finally identified as significant determinants of AF persistency and were used for further development of diagnostic models. Figure 2 illustrates the four identified variables (LAV, LVS extent, GMV, and APWD) which presented significant differences between paroxysmal and persistent AF cohorts both in training and validation sets. In addition, as the results of cv.glmnet are random, since the folds are selected at random. The randomness was reduced by running cv.glmnet for another five times to confirm the robustness in parameter selection, with the same parameters were reported in four runs misclassification error at 1-SE criteria: 0.201 ± 0.032, 0.191 ± 0.031, 0.212 ± 0.041, and 0.198 ± 0.032. Another run selected an extra parameter of LVS percentage at 1.0 mV besides the same aforementioned parameters, which failed to pass the subsequent logistic regression.

**TABLE 3:** Results from LASSO and logistic regression.

	LASSO regression	Logistic regression			
	Coefficient	*p*-value	OR	95% CI	
**Structure dimension**					
LAV	0.026	0.031	1.037	1.003	1.072
LAVI	0.021	0.119	1.056	0.986	1.132
**LVS extent dimension**					
LVS area at bipolar threshold 1.0 mV	0.020	<0.001	1.099	1.061	1.139
**Voltage dimension**					
GMV	-0.353	<0.001	0.249	0.151	0.41
**Non-invasive dimension**					
APWD	0.022	<0.001	1.101	1.07	1.133
**Integrated dimension**					
APWD	0.038	<0.001	1.069	1.036	1.103
LAV	0.018	<0.001	1.041	1.02	1.063
LVS area at bipolar threshold 1.0 mV	0.006	0.335	1.029	0.971	1.091
GMV	-0.164	0.431	0.689	0.273	1.740

LAV, left atrial volume; LAVI, left atrial volume index; LVS, low voltage substrate; GMV, global mean voltage; APWD, duration of the amplified digital P-wave.

#### 4.2.2. Integrated model

**FIGURE 3:**
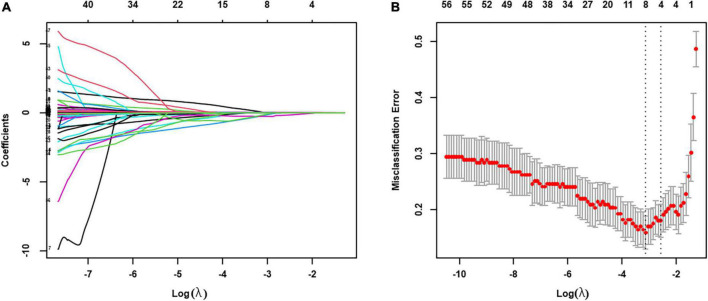
LASSO regression was used to select the relevant factors for AF persistency. **(A)** LASSO coefficient curves for the 62 clinical variables; **(B)** tuning parameter (λ) selection in the LASSO model used 10-fold cross-validation *via* minimum criteria. The partial likelihood deviance (binomial deviance) curve was plotted vs. log (λ). Dotted vertical lines were drawn at the optimal values by using the minimum criteria (λ value of 0.0438) and the one SE of the minimum criteria (the 1-SE criteria, λ value of 0.0765). The optimal value of λ (the vertical dashed line) for 10-fold cross-validation was used to select optimal variables. LASSO, least absolute shrinkage and selection operator.

The integrated model was developed by initially entering all 62 parameters from both electro-anatomical mapping and non-invasive APWD dimensions, which identified four parameters including APWD, LAV (without inclusion of PVs), LVS extent (absolute LVS area at bipolar threshold of 1.0 mV) and GMV (Figure 3). Subsequently, only APWD and LAV remained significant (*p* < 0.001) in multivariate logistic regression and were used to develop the bi-variate integrated model (APWD + LAV) for identification of persistent AF patients (Table 3).

### 4.3. Model evaluation in training set and validation set

#### 4.3.1. Discrimination between paroxysmal and persistent AF

##### 4.3.1.1. Comparison among diagnostic models from electro-anatomical parameters

**FIGURE 4:**
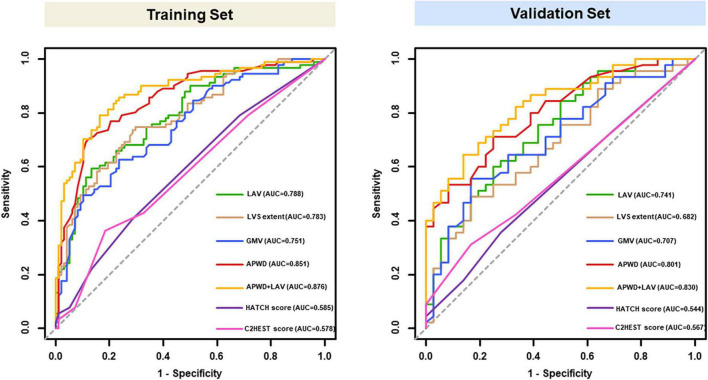
ROC curves of models for identification of AF persistency in training **(left)** and validation set **(right)**. Results of LAV (green), LVS extent (brown), GMV (blue) and APWD (red) represent the discriminative performance in single-dimension. The integrated (bi-dimensional) model (APWD + LAV) is illustrated in yellow curve. The HATCH score and C2HEST score were presented in purple and pink curves, respectively. ROC, receiver operating characteristics; AUC, area under the curve; LAV, left atrial volume; LVS, low voltage substrate; GMV, global mean voltage; APWD, duration of the amplified digital P-wave.

As illustrated in Figure 4, among the four single-dimension diagnostic models, APWD achieved the highest discriminative power both in training set (AUC: 0.851) and validation set (AUC: 0.801) for differentiating patients with paroxysmal from persistent AF type. Although LAV, LVS extent, and GMV presented rather good discriminative power in the training set (AUC: 0.788, 0.783, and 0.751, respectively), their discriminative power in validation set was attenuated (AUC: 0.741, 0.682, and 0.707, respectively). In contrast to GMV and LVS extent, LAV displayed a rather stable and superior discriminative power for AF subtype. The integrated model (incorporating APWD and LAV), achieved the highest discriminative performance in both sets among all diagnostic models (AUC: 0.876 and 0.830, respectively). Notably, in comparison to APWD alone, the addition of LAV did not significantly improve the discriminative power of integrated model (APWD + LAV) regarding AUC in training set (difference in AUC: 0.025, 95%CI: −0.003–0.054, *p* = 0.079) and validation set (difference in AUC: 0.029, 95%CI: −0.015–0.073, *p* = 0.193). However, incorporation of LAV to APWD conferred an increased accuracy in reclassifying individuals to proper AF subtypes than APWD alone in the training set (NRI: 0.642, 95%CI: 0.372–0.912, *p* < 0.001; IDI: 0.074, 95%CI: 0.035–0.113, *p* < 0.001) and validation set (NRI: 0.300, 95%CI: −0.129–0.729, *p* = 0.17; IDI: 0.047, 95%CI: 0.001–0.094, *p* = 0.044) (Supplementary Table 2). In order to provide further information on the robustness of results, we performed analysis with another four random splits of the original dataset by reporting the AUC of each model in each split. The selected parameters remained the same in four re-splits with minor variation in AUC (Supplementary Table 3).

##### 4.3.1.2. Discriminative performance of HATCH score and C2HEST score for AF persistency

As in Figure 4. Both HATCH score and C2HEST score, despite incorporation of multiple parameters, achieved poor discriminative performance for AF persistency in both training set (AUC: 0.585 for HATCH score, 0.578 for C2HEST score) and validation set (AUC: 0.544 for HATCH score, 0.567 for C2HEST score). Delong’s test showed significant difference in AUC between: APWD vs. HATCH score (*p* < 0.001 in both training and validation sets); APWD vs. C2HEST score (*p* < 0.001 in both training and validation sets).

#### 4.3.2. Calibration performance between predicted and observed probabilities for persistent AF

##### 4.3.2.1. Comparison among diagnostic models from electro-anatomical parameters

**FIGURE 5:**
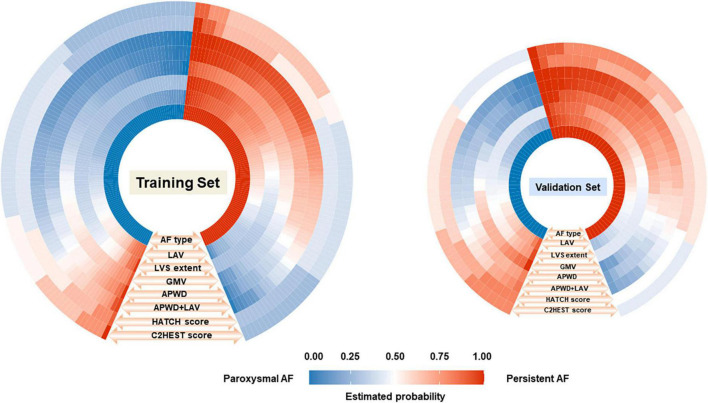
Comparison between the estimated probability for persistent AF by each model (seven outer layers) and the actual AF types (inner layer, ground truth) in training **(left)** and validation **(right)** sets. The figure consists of eight layers, the inner layer represents the ground truth AF types [paroxysmal (blue) or persistent AF (red)] of each individual (small rectangle), and the total number of rectangles in blue and red in the inner layer represents the number of patients in paroxysmal and persistent AF cohort, respectively. As illustrated by the orange arrows and labels at each layer (LAV, LVS extent, GMV, APWD, APWD + LAV, HATCH score, and C2HEST score) in an outward direction, the estimated probability for persistent AF of each individual (small rectangle) by each model was demonstrated in different grades of blue and red, indicating the probability for persistent AF ranging from “0” to “1.0.” The fifth (APWD) and sixth (APWD + LAV) layers which share a higher level of resemblance in color distribution to the inner layer, indicating a better calibration performance. LAV, left atrial volume; LVS, low voltage substrate; GMV, global mean voltage; APWD, duration of the amplified digital P-wave.

The estimated individual probabilities for persistent AF by each model are demonstrated in Figure 5. When compared with the actual AF subtype of each individual (inner layer), the four single-dimension diagnostic models (LAV, LVS extent, GMV, and APWD) displayed varying extent of accuracy among which APWD and LAV independently demonstrated the most consistent agreement to actual AF subtypes, both in training and validation sets. In addition, when combined (APWD + LAV), the integrated model (the sixth layer) further improved the diagnostic accuracy. Calibration plots of each model, in combination with Brier score, were used to illustrate the agreement between estimated and actual AF subtypes. As illustrated in Supplementary Figure 2, among single-dimension models in the training set, LAV and APWD displayed a good agreement between estimated and actual AF subtypes with Brier score of 0.188 and 0.157, respectively. LVS extent and GMV, however, displayed less satisfying agreement from calibration plots with Brier score of 0.193 and 0.202, respectively. In the validation set, on the other hand, only APWD maintained a good consistency between estimated and actual AF subtype (Brier score 0.180), whereas other single-dimension models from electro-anatomical mapping data displayed different extents of discrepancy (Brier score 0.203, 0.224, and 0.216 for LAV, LVS extent, and GMV, respectively).

##### 4.3.2.2. Calibration performance of HATCH score and C2HEST score for AF persistency

**FIGURE 6:**
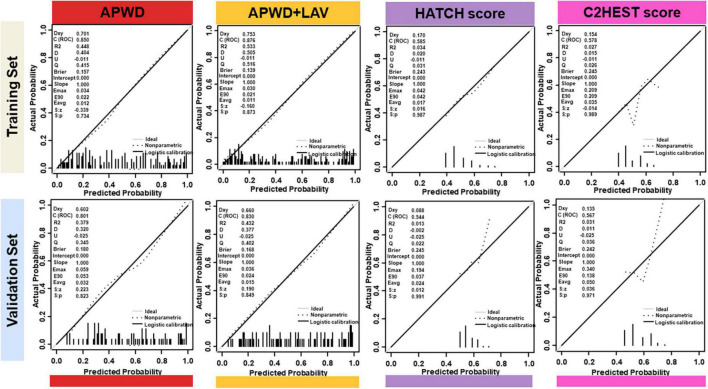
Calibration plots of integrated model (APWD + LAV), APWD, HATCH score and C2HEST score in training **(upper)** and validation **(lower)** sets. The diagonal gray line indicates perfect prediction of the ideal model. The dashed line represents the performance of the model, the dark solid line represents the calibrated model performance and being closer to the diagonal gray line indicated that the model has better prediction ability. In the left upper corner, different parameters are listed to provide more information of models including Dxy, ROC (equals to 1/2Dxy + 0.5), R2 and Brier Score. LAV, left atrial volume; APWD, duration of the amplified digital P-wave.

In contrast to aforementioned diagnostic models, HATCH score and C2HEST score demonstrated significant inconsistency with the actual AF types of patients in both training and validation sets, as in Figure 5. In addition, comparison among APWD, APWD + LAV, HATCH score and C2HEST score was displayed in Figure 6. In contrast to the good agreement observed in APWD and APWD + LAV (Brier score of 0.138 and 0.168 in training and validation sets), significant discrepancy was observed for HATCH score (Brier score of 0.243 and 0.245) and C2HEST score (Brier score of 0.245 and 0.242).

#### 4.3.3. Decision curve analysis (DCA) for net benefit assessment

**FIGURE 7:**
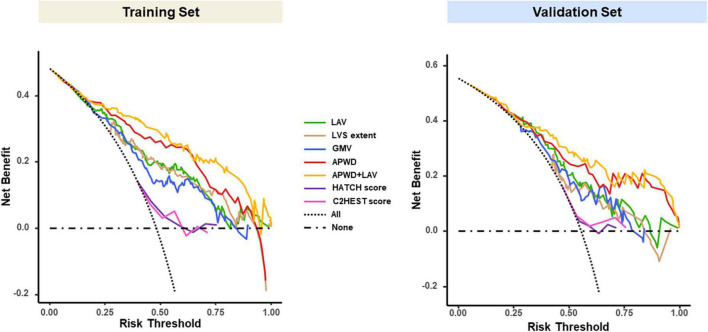
Decision curve analysis (DCA) of each model in training **(left)** and validation **(right)** sets. The vertical (Y) axis measured the net benefit, the horizontal (X) axis represents the range of potential risk threshold for persistent AF from diagnostic models. The “All” dashed dark line represented the assumption that all patient has persistent AF. The “None” dark dashed line represented the assumption that no patient has persistent AF. The clinical usefulness of each model across the range of risk thresholds is illustrated in different colors: green for LAV, brown for LVS extent, blue for GMV, red for APWD, yellow for APWD + LAV. The HATCH score and C2HEST score were presented in purple and pink curves, respectively. The model which positions closer to the right upper corner indicates better clinical usefulness. LAV, left atrial volume; LVS, low voltage substrate; GMV, global mean voltage; APWD, duration of the amplified digital P-wave.

The DCA curves of each model in both training and validation sets (Figure 7) demonstrated that APWD alone was associated with significantly higher levels of net benefit in contrast to other single-dimension models across a range of underlying thresholds. Moreover, when combined with LAV, the associated increment in net benefit was more profound in training set than in validation set, indicating that APWD alone could facilitate identification of individuals with persistent AF. HATCH score and C2HEST score, on the other hand, demonstrated only marginal net benefit with the reference lines of “All” and “None.”

#### 4.3.4. Diagnostic thresholds of identified predictors for AF persistency

##### 4.3.4.1. Diagnostic performance of diagnostic models from electro-anatomical parameters

**FIGURE 8:**
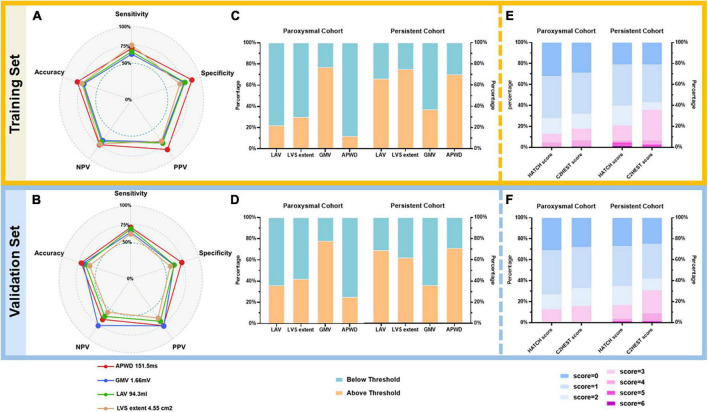
Diagnostic performance of models using respective optimal thresholds in training **(A,C,E)** and validation **(B,D,F)** sets. In the left panel **(A,B)**, a pentagon is generated for training and validation sets, respectively, to illustrate the diagnostic performance of each model using respective thresholds as listed on the legend at bottom. Every pentagon contains three reference levels (dashed circles), representing 50, 75, and 100%, respectively. The middle panel **(C,D)** illustrates the percentage of individuals above (orange) or below (emerald) respective AF-persistency thresholds of each model in paroxysmal and persistent cohorts. For LAV, LVS extent, and APWD, individuals above respective thresholds are diagnosed as positive for persistent AF, whereas for GMV, those below the threshold are diagnosed as positive for persistent AF. In the right panel **(E,F)**, the diagnostic performance of HATCH score and C2HEST score is illustrated as stacked barplots, the percentage of different scores is displayed as bars from blue (lowest risk) to purple (highest risk) with 1-score increment, as the legends at the bottom. LAV, left atrial volume; LVS, low voltage substrate; GMV, global mean voltage; APWD, duration of the amplified digital P-wave; NPV, negative predictive value; PPV, positive predictive value.

As displayed in Figures 8A–D, the optimal threshold of each model was determined using Youden index based on the ROC curve of the training set (APWD threshold: 151.5 ms; LAV (94.3 mL); LVS extent at 1.0 mV: 4.55 cm^2^; GMV: 1.66 mV), as shown in Table 4. In the training set, among single-dimension models, APWD with a threshold of 151.5 ms displayed an accuracy of 79.4%, with sensitivity of 70.3%, specificity of 87.8%, PPV of 84.2% and NPV of 76.1%. Other models, on the other hand, demonstrated less satisfying diagnostic performance using respective thresholds. In the validation set, when applying the thresholds determined from training set, all models displayed weakened diagnostic power. Nevertheless, APWD maintained the best performance (accuracy of 72.8% with sensitivity of 71.1%, specificity of 75.0%, PPV of 78% and NPV of 72.8%) among all single-dimension diagnostic models.

**TABLE 4:** Diagnostic thresholds of identified predictors for AF persistency.

Parameter (threshold)	sensitivity	specificity	PPV	NPV	Accuracy
**Training set**					
APWD (151.5 ms)	70.3%	87.8%	84.2%	76.1%	79.4%
LAV (94.3 mL)	65.9%	77.6%	73.2%	71.0%	72.0%
LVS extent (4.55 cm^2^)	74.7%	70.4%	70.1%	75.0%	72.5%
GMV (1.66 mV)	62.5%	76.5%	71.3%	68.8%	69.8%
**Validation set**					
APWD (151.5 ms)	71.1%	75.0%	78.0%	67.5%	72.8%
LAV (94.3 mL)	68.9%	63.9%	70.5%	62.2%	66.7%
LVS extent (4.55 cm^2^)	62.2%	58.3%	65.1%	55.3%	60.5%
GMV (1.66 mV)	64.4%	63.6%	78.4%	77.8%	70.4%

PPV, positive predictive value; NPV, negative predictive value; APWD, duration of the amplified digital P-wave; LAV, left atrial volume; LVS, low voltage substrate; GMV, global mean voltage.

**FIGURE 9:**
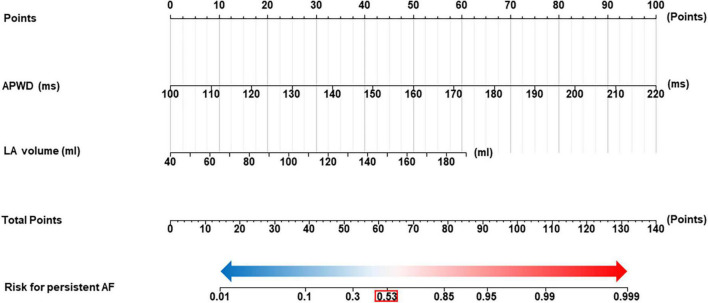
Nomogram for identification of individuals with persistent AF. The nomogram was developed using integrated model (APWD + LAV) in the training set. The total score of the nomogram for individual is the sum of the corresponding score assigned to each risk factor, and the total score corresponds to the individual risk for persistent AF. The optimal cut-off value for the estimated risk for persistent AF in the nomogram is 0.53 (highlighted by the red box), individuals who are estimated with risk higher than 0.53 are diagnosed as high probability for AF persistency and those below 0.53 as high probability for paroxysmal Af type. LAV, left atrial volume; duration of the amplified digital P-wave.

Additionally, based on the robust performance of the integrated model (APWD + LAV), we proposed a nomogram to facilitate a more accurate estimation of the individual risk for persistent AF. Using the coordinates of the ROC curve of the integrated model in the training set, an estimated risk of 0.53 was determined in the nomogram to classify the risk range into low risk (below 0.53) and high risk (above 0.53), as illustrated in Figure 9.

##### 4.3.4.2. Diagnostic performance of HATCH score and C2HEST score

As shown in Figures 8E, F, the diagnostic performance of HATCH score and C2HEST score was evaluated from minimum point (lowest risk) to maximum points (highest risk). As a result, both HATCH score and C2HEST score failed to demonstrate a clear difference between patients with paroxysmal and persistent AF.

## 5. Discussion

The current study identified four major electrophysiological and structural factors that are involved in LA remodeling associated with paroxysmal or persistent AF type. Analysis of 62 LA structure- and electrophysiology-related parameters revealed a determinant role of only four factors that are associated with AF persistency: (1) duration of bi-atrial conduction time (as identified by APWD analysis in the digital 12-lead-ECG), (2) LA volume, (3) extent of LA LVS and (4) LA GMV. The study identifies for the first time the “critical thresholds” for prolonged bi-atrial conduction time, increased LA volume, LA low voltage extent, LA mean voltage that are associated with persistent AF type. Among these four factors, the best predictors of AF persistency are (1) APWD > 151 ms (AUC: 0.85) followed by (2) LA volume > 94 mL (AUC: 0.79). The combination of APWD and LAV achieved the highest AUC (0.88) for identifying patients with persistent AF type.

In summary, the study identifies thresholds for the main electrical (APWD) and structural (LAV) factors of LA remodeling, that are associated with progression of paroxysmal to persistent AF type.

### 5.1. Atrial fibrillation progression

“Atrial fibrillation begets AF” was a concept brought up by Wijffels et al. to describe the self-sustaining nature of AF due to reduction of atrial refractory period and delayed conduction (21). Progression from paroxysmal to persistent AF led to a consequence of increased difficulty to restore and maintain SR and higher risk for heart failure, thromboembolism and mortality (7, 10, 22). In an effort to facilitate decision-making for early intervention and further reduce the morbidity and mortality burdened by AF progression, several studies were carried out to unravel the relevant predictors and underlying mechanisms. Results from 10-year follow-up of CRAF (Canadian Registry of AF) reported that increasing age and structural predictors including mitral regurgitation and LA dilation were significant predictors for progression from paroxysmal to persistent AF (8). Pertinent studies by Blum et al. identified higher age and hypertension were positively associated with AF-progression rate (7, 23). The HATCH score was developed by de Vos et al. to estimate the probability for AF-progression using similar components as in the CHA2DS2-Vasc Score (10). However, although the HATCH score outperformed other single predictors in its components, the AUC was only 0.675 and the score was more oriented at age and underlying heart disease whereas little information was provided regarding the actual atrial remodeling status like atrial enlargement and atrial conduction impairment (10). Another study with 12-year follow-up using the HATCH score reported C statistics of 0.6 to predict AF-progression (24). Nevertheless, electrophysiological parameters of LA remodeling with regard to AF progression have not yet been systematically studied.

### 5.2. Feature-based diagnostic models incorporating intra-cardiac mapping and 12-lead-ECG data enable identification of multi-factorial determinants of AF-progression

#### 5.2.1. Left atrial volume as marker of atrial structural macro-remodeling

Left atrial enlargement, as a predominant macroscopic manifestation for structural remodeling, was a significant predictor for progression from paroxysmal to persistent AF. Akutsu et al. reported that LA diameter (LAD) > 40 mm was associated with a 3.82-fold increase of risk for AF-progression (25). Similar findings (Odds Ratio: 2.29 for LAD > 40 mm) were also reported by Koide et al. in a larger cohort (26). However, there are certain restrictions to those findings: First, in contrast to LA volume or LA strain which represent the global LA size and function, LAD evaluates LA size in a single spatial dimension and is therefore not an ideal parameter for assessment of macroscopic structural LA remodeling. Second, as PVs have been identified as pivotal arrhythmogenic trigger sites in AF, their impact might have been underestimated in previous studies, which excluded PVs from the analysis of LA structural remodeling and focused on the LA body. Instead, a region-specific analysis, including the volume or surface area of the PVs might reveal additional insight (27). In the current study, we extracted data from high-definition electro-anatomical mapping during SR: A total of 62 parameters were extracted from the electro-anatomical maps and the 12-lead-ECG (Table 2). Sequential statistical testing revealed that absolute LA volume (LAV) was the best discriminator (among 11 other markers of LA size that were derived from the endocardial maps, as in Table 2) between paroxysmal and persistent AF type. Notably, the indexed LAV “LAVI” was less accurate than for discrimination between AF types. A potential explanation for that may be the following: irrespective of the individual patient’s BMI or BSA, an increase in the absolute LA volume may better represent the increase in atrial mass and a higher probability for harboring and maintaining reentry circuits during AF as an arrhythmogenic factor.

#### 5.2.2. Left atrial voltage reduction as a marker of cardiomyocyte death and fibrotic remodeling in atrial cardiomyopathy

Progression of paroxysmal to persistent AF has also been reported to involve increasing degrees of atrial fibrotic remodeling (13), constituting a key element of ACM (28). A number of clinical mapping studies in patients with AF have shown presence of LA low voltage areas (29–31) and prolonged fractionated potentials (PFP) or atrial late potentials (ALP) within/adjacent to LVS, representing areas of fibrotic remodeling with slow conduction (32, 33). Notably, persistent AF patients with LA LVS present a more advanced stage of ACM and have significantly higher (50%) arrhythmia recurrence rates at 12 months after PVI than patients without LVS (30% arrhythmia recurrence) (29, 30). Several studies have independently reported a specific distribution patterns of LA LVS during SR with antero-septal LA being most frequently impacted, followed by extension of LVS in later disease stages to the LA roof and LA posterior wall (14, 34). Because of this inhomogeneous development of LVS, we conducted different methods to assess the extent of global LA LVS: The extent of LVS was quantified (1) at < 0.5 mV and (2) < 1.0 mV threshold during SR. Moreover, the GMV as well as the regional mean bipolar voltages were assessed to identify the most important voltage-related marker that is associated with AF persistency: The two best indicators were found to be (1) extent of LVS at 1.0 mV threshold in SR and (2) LA GMV. However, their diagnostic performance for identification of persistent AF type remained significantly inferior to APWD or LAV.

#### 5.2.3. Duration of the amplified digital P-wave (APWD) as a non-invasive marker of bi-atrial conduction time discriminates paroxysmal from persistent AF with high accuracy

P-wave indices (PWI), due to their non-invasive nature and cost-effectiveness, have been favored in clinical practice as a screening tool. Akutsu et al. reported that the maximum PWD from standard 12-lead ECG was an independent predictor (HR: 5.49, *p* < 0.001) for progression to persistent AF after 12.9-year follow-up, whereas p-wave dispersion (Pd) was not significant in multivariate analysis (25). In contrast, Koide et al observed that Pd was the only significant AF-progression predictor among P-terminal force and PWD (26). Nevertheless, previous studies using PWI were predominantly based on results from the standard 12-lead ECG, and the accuracy of measurement was susceptible to ECG quality and algorithm efficacy. We recently introduced a novel method for measurement of PWD, which provides high correlation to the invasively measured bi-atrial conduction time during SR: The “duration of the amplified digital P-wave in the 12-lead-ECG during SR” was shown to correlate well with both the invasively measured bi-atrial conduction time and the extent of LA LVS (15). We therefore used the previously validated APWD in the current study to assess its diagnostic value for differentiating between the AF types. Although APWD represents a non-invasive parameter, it achieved the highest AUC (0.859 and 0.843 in training and validation sets, respectively) among all other single-dimension models. The electrophysiological feature of PWD actually represents different aspects of atrial remodeling, which are incorporated in the “time domain” of p-wave: both structural remodeling processes (atrial dilation and fibrotic remodeling with increased LVS formation and decrease of mean atrial voltage as demonstrated in the current study) implicate a prolongation of bi-atrial conduction time. Therefore, the “single” non-invasive parameter APWD reflects both the prolongation of atrial conduction time due to (1) LA dilation and (2) slow conduction because of increasing fibrotic remodeling. Therefore, it is explainable why APWD is the best diagnostic parameter for detection of atrial remodeling, ACM progression and as a result is well suited to detect differences in atrial remodeling that are associated with transition from paroxysmal to persistent AF.

Albeit the current model based on APWD is not able to provide specific information about which remodeling mechanism is dominant (LA dilation or LA fibrosis/slow conduction), it allows detection of advanced ACM and atria remodeling that play key role as underlying substrate for transition from paroxysmal to persistent AF.

#### 5.2.4. HATCH score and C2HEST score: What are the limitations?

HATCH score and C2HEST score consist of clinical parameters and were originally developed to predict future events of AF progression and incident AF, respectively (10, 11). Although, several studies have reported their varying accuracy for different clinical endpoints, little is known about their performance for identifying the remodeling discrepancies between paroxysmal and persistent AF (35–37). In current study, both scoring systems achieved poor performance regarding discrimination (AUC < 0.60), calibration and net benefit. However, they excelled in FHS (38), CHARGE-AF score (39) and ARIC score (40) in practicability by using less and convenient parameters, the components do not reflect the pathological manifestation of atrial remodeling (e.g., atrial dilation and voltage reduction…). As the presence of hypertension, heart failure, COPD and older age were frequently reported as common risk factors in various cardio-cerebral-vascular diseases, their accuracy in identifying atrial remodeling evolution were therefore unsurprisingly inadequate, despite the statistical adjustments made to assign a weighted score. Moreover, the development of HATCH score and C2HEST score share analogous limitations as they were derived from databases of the large survey (The Euro Heart Survey on AF) and the insurance system (Chinese Yunnan Insurance Database), the individual comorbidities therefore remained as the major data source for model development. In contrast, as the current study provided 62 parameters (47 from high density electro-anatomical mapping and 15 non-invasive parameters), a more comprehensive evaluation of remodeling discrepancies between paroxysmal and persistent AF was enabled. As a result, APWD alone or in combination with LAV (the integrated model), a robust performance was achieved with even better practicability than scoring systems. Another explanation would be that the enrolled patients in our study had significant low prevalence of Chronic Obstructive Pulmonary Disease (COPD) and absence of hyperthyroidism (which is a contra-indication to invasive LA mapping for PVI), which also may have contributed to the limited efficacy of HATCH score and C2HEST score in current study.

## 6. Conclusion

The current study identified four major electrophysiological and structural factors (among 62 analyzed factors) that are involved in LA remodeling and advanced ACM, determining the clinical AF type “paroxysmal” or “persistent”: (1) duration of bi-atrial conduction time (as identified by APWD analysis in digital 12-lead-ECG), (2) LA volume, (3) extent of LA LVS and (4) LA GMV. In addition, we identified for the first time the “critical thresholds” for prolonged bi-atrial conduction time (as determined by APWD in 12-lead-ECG), increased LA volume, LA low voltage extent, LA mean voltage that are associated with persistent AF type. Among these four factors, the best predictors of AF persistency are (1) APWD > 151 ms (AUC: 0.85), followed by (2) LA volume > 94 mL (AUC: 0.79). The combination of APWD and LAV achieved the highest AUC (0.88) for identifying patients with persistent AF type.

## Data availability statement

The raw data supporting the conclusions of this article will be made available by the authors, without undue reservation.

## Author contributions

AJ, B-ME, and TA mapped the study patients. DN extracted the parameters from CARTO maps. TH and DN performed the machine-learning algorithm and statistical analyses. TH and other authors collected baseline demographic data. TH and AJ measured the APWD blinded to patients’ characteristics. TH drafted the manuscript. AJ, AL, and TA revised the manuscript. All authors contributed to the article and approved the submitted version.

## Footnotes

1
http://www.r-project.org/
