# Supplementary material for: Structural and electrophysiological determinants of atrial cardiomyopathy identify remodeling discrepancies between paroxysmal and persistent atrial fibrillation
Source: Front Cardiovasc Med. 2023 Jan 11;9:1101152. doi: 10.3389/fcvm.2022.1101152 (PMC9874680; doi:10.3389/fcvm.2022.1101152)
Supplement: Supplementary file 1 [file Data_Sheet_1.docx]

**Structural and electrophysiological determinants of atrial cardiomyopathy identify remodeling discrepancies between paroxysmal and persistent atrial fibrillation**

**Supplemental Appendix**

**Supplemental tables (Page 2 - 7)**

**Supplemental figure (Page 8 - 9)**

**Supplemental Table 1.** **Electro-anatomical characteristics between training and validation set**

|  | Training Set (n=189) | Validation Set (n=81) | P value |
| --- | --- | --- | --- |
| **LVS Extent dimension** | | | |
| LVS Area with PV (0.5mV). (cm^2^) | 38.34 ± 22.02 | 43.15 ± 22.41 | 0.071 |
| LVS Area without PV (0.5mV). (cm^2^) | 3.71 ± 8.5 | 4.14 ± 8.32 | 0.860 |
| LVS Area with PV (1mV). (cm^2^) | 62.44 ± 28.64 | 68.25 ± 29.33 | 0.107 |
| LVS Area without PV (1mV). (cm^2^) | 11.16 ± 14.55 | 12.01 ± 14.93 | 0.866 |
| LVS percentage without PV (0.5mV). (%) | 5.59 ± 12.35 | 5.9 ± 11.6 | 0.922 |
| LVS percentage with PV (0.5mV). (%) | 25.35 ± 14.73 | 27.31 ± 14.72 | 0.215 |
| LVS percentage without PV (1mV). (%) | 16.74 ± 21.41 | 17.08 ± 20.74 | 0.980 |
| LVS percentage with PV (1mV). (%) | 41.29 ± 18.61 | 43.17 ± 18.62 | 0.431 |
| **Structural dimension** | | | |
| LA Volume with PV. (ml) | 141.61 ± 33.16 | 148.19 ± 34.08 | 0.131 |
| LA Volume without PV. (ml) | 93.13 ± 24.34 | 98.03 ± 27.1 | 0.182 |
| LA Volume Index with PV. (ml/m^2^) | 72.06 ± 16.06 | 74.12 ± 17.09 | 0.345 |
| LA Volume Index without PV. (ml/m^2^) | 47.45 ± 12.15 | 49.09 ± 13.83 | 0.330 |
| PV Volume. (ml) | 48.47 ± 12.86 | 50.16 ± 12.41 | 0.319 |
| PV Volume Index. (ml/m^2^) | 24.62 ± 5.93 | 25.03 ± 6.02 | 0.602 |
| LA Area with PV. (cm^2^) | 152.55 ± 25.63 | 159.72 ± 27.99 | 0.087 |
| LA Area without PV. (cm^2^) | 66.53 ± 12.32 | 70.02 ± 13.5 | 0.025 |
| LA Area Index with PV. (cm^2^/m^2^) | 77.53 ± 11.14 | 79.52 ± 11.55 | 0.183 |
| LA Area Index without PV. (cm^2^/m^2^) | 33.89 ± 5.96 | 34.99 ± 6.74 | 0.183 |
| PV Area. (cm^2^) | 86.02 ± 19.11 | 89.71 ± 20.40 | 0.156 |
| PV Area Index. (cm^2^/m^2^) | 43.64 ± 8.51 | 44.54 ± 8.55 | 0.429 |
| **Voltage dimension. (mV)** | | | |
| Bipolar LA Global Median Voltage without PV | 2.11 ± 0.84 | 2.15 ± 0.87 | 0.765 |
| Bipolar LA Global Mean Voltage without PV | 1.85 ± 0.79 | 1.85 ± 0.90 | 0.987 |
| Bipolar LA Global Mean Voltage with PV | 1.48 ± 0.57 | 1.46 ± 0.6 | 0.844 |
| Bipolar LA Global Median Voltage with PV | 1.22 ± 0.55 | 1.2 ± 0.56 | 0.700 |
| Bipolar LA Maximum Voltage without LAA | 10.23 ± 3.66 | 10.92 ± 3.82 | 0.161 |
| Bipolar LA Maximum Voltage with LAA | 7.85 ± 4.24 | 8.54 ± 3. 82 | 0.209 |
| Bipolar LA Anterior Mean Voltage | 1.76 ± 0.96 | 1.83 ± 1.13 | 0.945 |
| Bipolar LA Posterior Mean Voltage | 2.17 ± 1.19 | 2.1 ± 1.12 | 0.856 |
| Bipolar LA Inferior Mean Voltage | 1.81 ± 0.77 | 1.76 ± 0.79 | 0.563 |
| Bipolar LA Lateral Mean Voltage | 2.2 ± 0.91 | 2.36 ± 1.02 | 0.422 |
| Bipolar LAA Mean Voltage | 3.05 ± 1.5 | 3.27 ± 1.36 | 0.204 |
| Bipolar Left PV Mean Voltage | 0.78 ± 0.5 | 0.74 ± 0.44 | 0.703 |
| Bipolar Right PV Mean Voltage | 1 ± 0.48 | 0.9 ± 0.47 | 0.159 |
| Bipolar LA Anterior Median Voltage | 1.6 ± 0.91 | 1.71 ± 1.09 | 0.746 |
| Bipolar LA Posterior Median Voltage | 1.99 ± 1.14 | 1.95 ± 1.09 | 0.956 |
| Bipolar LA Inferior Median Voltage | 1.63 ± 0.72 | 1.6 ± 0.76 | 0.720 |
| Bipolar LA Lateral Median Voltage | 2.06 ± 0.93 | 2.2 ± 1.01 | 0.495 |
| Bipolar LAA Median Voltage | 2.95 ± 1.52 | 3.16 ± 1.34 | 0.209 |
| Bipolar Left PV Median Voltage | 0.52 ± 0.48 | 0.48 ± 0.35 | 0.988 |
| Bipolar Right PV Median Voltage | 0.73 ± 0.46 | 0.64 ± 0.42 | 0.120 |
| Bipolar LA Anterior Maximum Voltage | 6.54 ± 3.28 | 6.41 ± 3.42 | 0.803 |
| Bipolar LA Posterior Maximum Voltage | 8.04 ± 3.67 | 7.74 ± 3.68 | 0.488 |
| Bipolar LA Inferior Maximum Voltage | 7.78 ± 3.24 | 7.68 ± 3.41 | 0.531 |
| Bipolar LA Lateral Maximum Voltage | 7.51 ± 3.01 | 8.05 ± 3.2 | 0.399 |
| Bipolar LAA Maximum Voltage | 7.69 ± 3.71 | 8.5 ± 3.81 | 0.118 |
| Bipolar Left PV Maximum Voltage | 7.58 ± 4.53 | 7.57 ± 4.01 | 0.984 |
| Bipolar Right PV Maximum Voltage | 7.76 ± 6.46 | 6.98 ± 3.36 | 0.670 |

LVS, low voltage substrate; PV, pulmonary vein; LA, left atrial; LAA, left atrial appendage

**Supplemental Table 2. Comparison of AUC, IDI and NRI between APWD and (APWD+LAV)**

|  | Training Set | | | | Validation Set | | | |
| --- | --- | --- | --- | --- | --- | --- | --- | --- |
| APWD vs. APWD+LAV | value | 95%CI | | p value | value | 95%CI | | p value |
| Difference in AUC | 0.026 | -0.003 | 0.054 | 0.079 | 0.029 | -0.015 | 0.073 | 0.193 |
| IDI | 0.074 | 0.035 | 0.113 | <0.001 | 0.047 | 0.001 | 0.094 | 0.044 |
| NRI | 0.642 | 0.372 | 0.912 | <0.001 | 0.300 | -0.129 | 0.729 | 0.170 |

**Supplemtanl Table 3. AUC of each model from four random re-splits of the original dataset**

| **First Re-split** | | | | | |
| --- | --- | --- | --- | --- | --- |
| **Set** | Model | AUC | Std. Error | 95% Confidence Interval | |
|  | LVS extent | 0.759 | 0.034 | 0.691 | 0.826 |
|  | LAV | 0.776 | 0.034 | 0.71 | 0.842 |
|  | GMV | 0.708 | 0.037 | 0.635 | 0.781 |
| **Training Set** | APWD | 0.85 | 0.028 | 0.795 | 0.905 |
|  | APWD+LAV | 0.868 | 0.026 | 0.817 | 0.919 |
|  | HATCH score | 0.602 | 0.041 | 0.521 | 0.683 |
|  | C2HEST score | 0.583 | 0.042 | 0.502 | 0.665 |
|  | LVS extent | 0.696 | 0.058 | 0.593 | 0.812 |
|  | LAV | 0.706 | 0.059 | 0.58 | 0.818 |
|  | GMV | 0.697 | 0.058 | 0.583 | 0.811 |
| **Validation Set** | APWD | 0.799 | 0.048 | 0.705 | 0.893 |
|  | APWD+LAV | 0.818 | 0.046 | 0.727 | 0.908 |
|  | HATCH score | 0.552 | 0.064 | 0.427 | 0.677 |
|  | C2HEST score | 0.578 | 0.063 | 0.454 | 0.702 |
| **Second Re-split** | | | | | |
| **Set** | Model | Area | Std. Error | 95% Confidence Interval | |
|  | LVS extent | 0.742 | 0.038 | 0.668 | 0.816 |
|  | LAV | 0.765 | 0.037 | 0.693 | 0.837 |
|  | GMV | 0.692 | 0.041 | 0.613 | 0.772 |
| **Training Set** | APWD | 0.837 | 0.031 | 0.775 | 0.898 |
|  | APWD+LAV | 0.853 | 0.029 | 0.796 | 0.911 |
|  | HATCH score | 0.577 | 0.044 | 0.49 | 0.664 |
|  | C2HEST score | 0.551 | 0.045 | 0.463 | 0.638 |
|  | LVS extent | 0.741 | 0.048 | 0.647 | 0.836 |
|  | LAV | 0.731 | 0.05 | 0.634 | 0.828 |
|  | GMV | 0.724 | 0.05 | 0.626 | 0.821 |
| **Validation Set** | APWD | 0.828 | 0.039 | 0.751 | 0.905 |
|  | APWD+LAV | 0.855 | 0.036 | 0.785 | 0.926 |
|  | HATCH score | 0.602 | 0.055 | 0.493 | 0.71 |
|  | C2HEST score | 0.63 | 0.054 | 0.523 | 0.737 |
| **Third Re-split** | | | | | |
| **Set** | Model | Area | Std. Errorb | 95% Confidence Interval | |
|  | LVS extent | 0.762 | 0.034 | 0.695 | 0.83 |
|  | LAV | 0.775 | 0.034 | 0.709 | 0.841 |
|  | GMV | 0.707 | 0.037 | 0.634 | 0.781 |
| **Training Set** | APWD | 0.836 | 0.029 | 0.779 | 0.893 |
|  | APWD+LAV | 0.862 | 0.027 | 0.809 | 0.915 |
|  | HATCH score | 0.604 | 0.041 | 0.523 | 0.684 |
|  | C2HEST score | 0.582 | 0.041 | 0.501 | 0.664 |
|  | LVS extent | 0.701 | 0.057 | 0.588 | 0.813 |
|  | LAV | 0.686 | 0.059 | 0.571 | 0.802 |
|  | GMV | 0.701 | 0.058 | 0.587 | 0.814 |
| **Validation Set** | APWD | 0.826 | 0.045 | 0.738 | 0.914 |
|  | APWD+LAV | 0.837 | 0.044 | 0.751 | 0.922 |
|  | HATCH score | 0.547 | 0.065 | 0.42 | 0.673 |
|  | C2HEST score | 0.581 | 0.063 | 0.457 | 0.705 |
| **Fourth Re-split** | | | | | |
| **Set** | Model | Area | Std. Errorb | 95% Confidence Interval | |
|  | LVS extent | 0.778 | 0.034 | 0.712 | 0.843 |
|  | LAV | 0.785 | 0.034 | 0.718 | 0.851 |
|  | GMV | 0.726 | 0.037 | 0.654 | 0.798 |
| **Training Set** | APWD | 0.838 | 0.03 | 0.779 | 0.896 |
|  | APWD+LAV | 0.871 | 0.026 | 0.819 | 0.923 |
|  | HATCH score | 0.609 | 0.042 | 0.527 | 0.69 |
|  | C2HEST score | 0.59 | 0.042 | 0.507 | 0.672 |
|  | LVS extent | 0.668 | 0.058 | 0.554 | 0.782 |
|  | LAV | 0.68 | 0.057 | 0.568 | 0.793 |
|  | GMV | 0.661 | 0.059 | 0.546 | 0.776 |
| **Validation Set** | APWD | 0.828 | 0.043 | 0.744 | 0.912 |
|  | APWD+LAV | 0.823 | 0.044 | 0.737 | 0.908 |
|  | HATCH score | 0.541 | 0.062 | 0.419 | 0.662 |
|  | C2HEST score | 0.564 | 0.062 | 0.443 | 0.685 |

**Supplemental Figure 1. Definition of Left Atrial Segmentation, Pulmonary Vein Ostia and Antra**


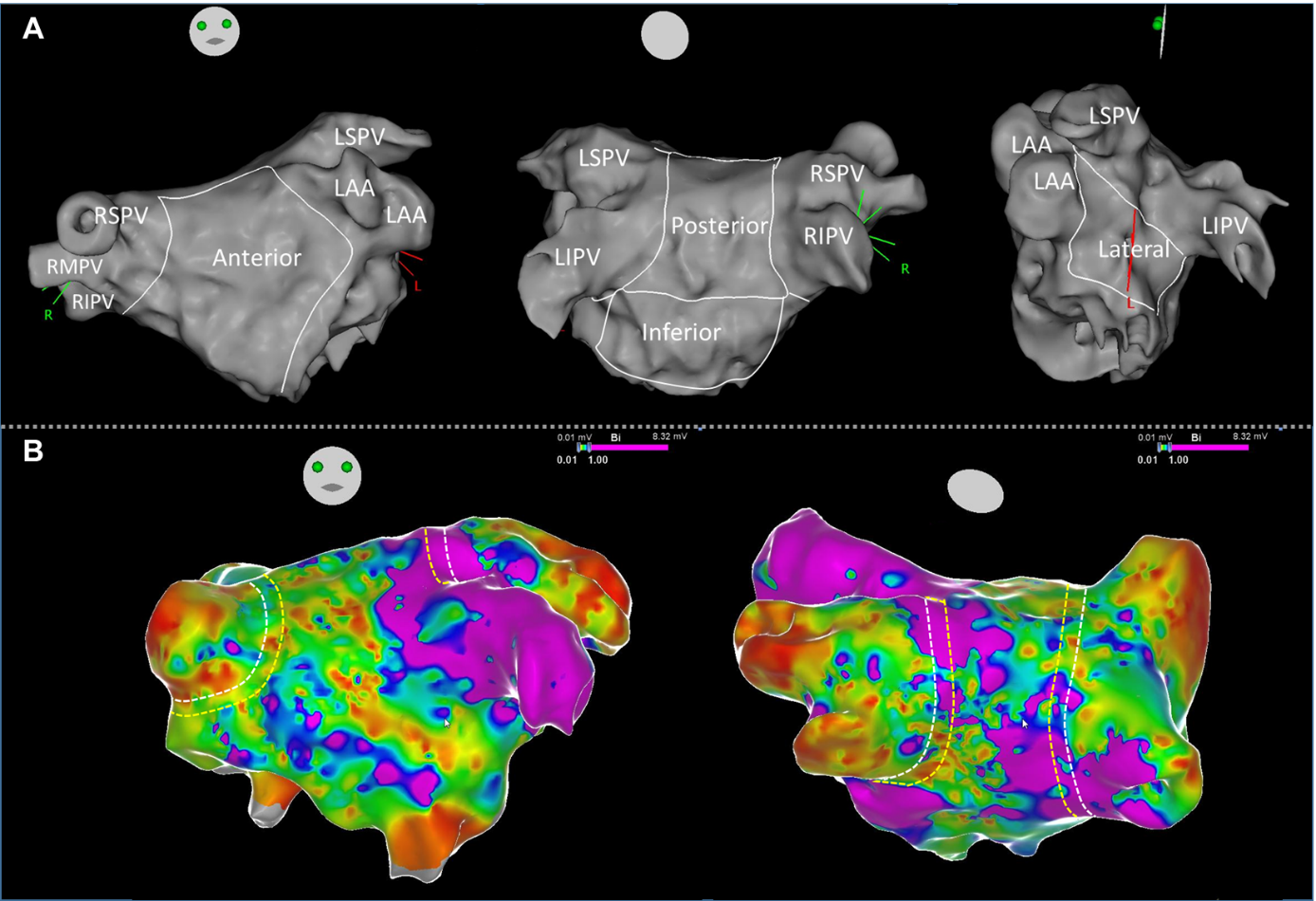


Supplemental Figure. Panel 1A illustrates the regional segmentation of left atrium in current study. Panel 1B illustrates the PV areas as defined by their ostia (white dashed lines) and their 5mm surrounding antral area (yellow dashed lines). PV, pulmonary vein; RSPV, right superior pulmonary vein; RMPV, right middle pulmonary vein; RIPV, right inferior pulmonary vein; LSPV, left superior pulmonary vein; LIPV, left inferior pulmonary vein; LAA, left atrial appendage.

**Supplental Figure 2. Calibration plots of selected predictor of respective dimension**


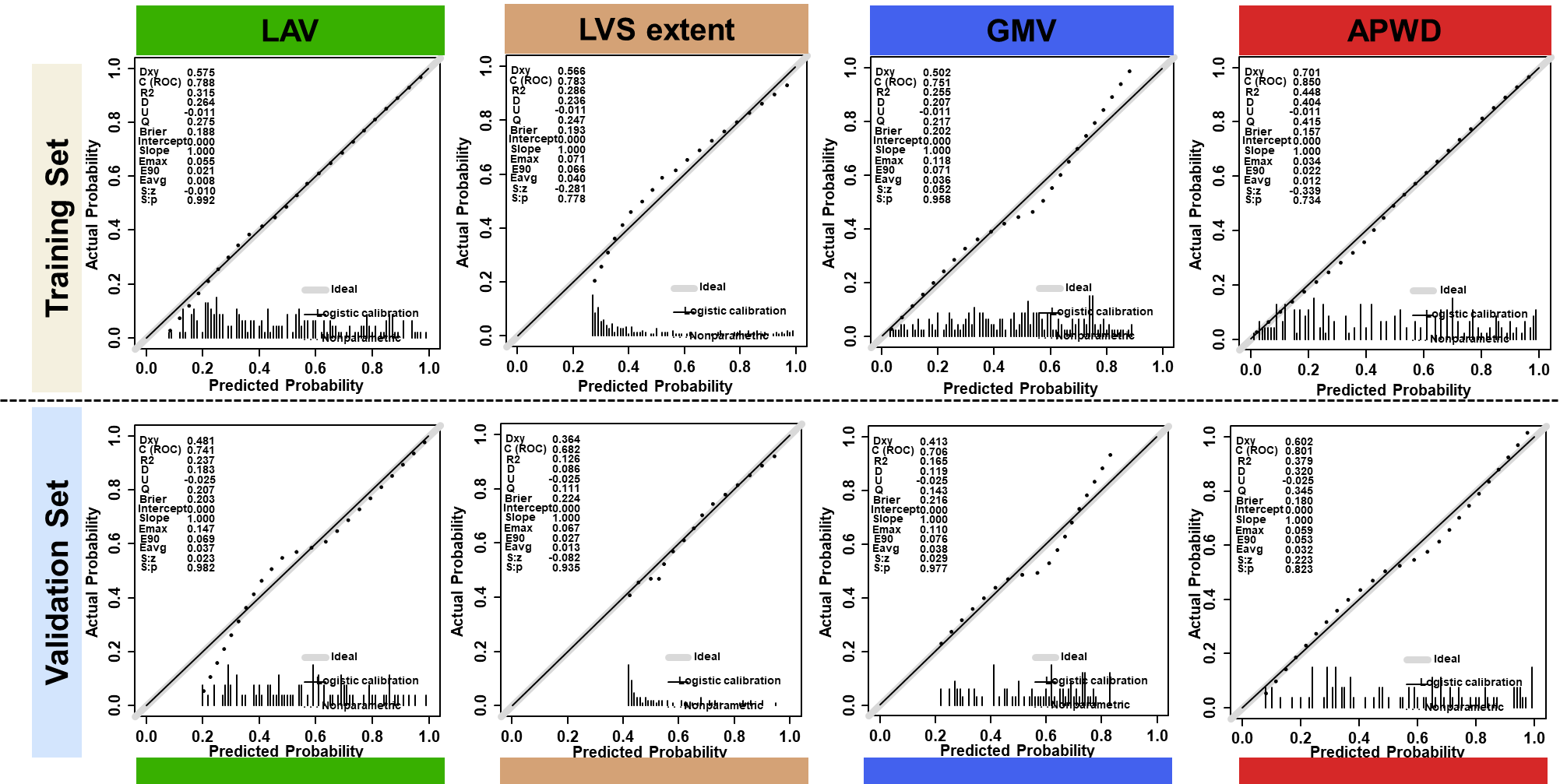


Supplemental Figure 2. Calibration plots of selected predictor (LAV, LVS extent, GMV and APWD) for AF-persistency in training (upper) and validation (lower) sets. The diagonal gray line indicates perfect prediction of the ideal model. The dashd line representes the performance of the model, the dark solid line represents the calibrated model performance and being closer to the diagonal gray line indicated that the model has better prediction ability. In the left upper corner, different parameters are listed to provide more information of models including Dxy, ROC (equals to 1/2Dxy+0.5),R2 and Brier Score. LAV, left atrial volume; LVS, low voltage substrate; GMV, global mean voltage; APWD, duration of amplified digital p-wave.
